# Single-Institution Outcomes of Neoadjuvant Gemcitabine and Nab-Paclitaxel Combination in Borderline Resectable Pancreatic Adenocarcinoma: A Three-Year Retrospective Audit

**DOI:** 10.7759/cureus.108823

**Published:** 2026-05-13

**Authors:** Goutham Reddy Ramidi, Rajan Yadav, Abinash Patnaik, Goutham Sunny

**Affiliations:** 1 Medical Oncology, R.V.M Institute of Medical Sciences and Research Center, Hyderabad, IND; 2 Medical Oncology, Gujarat Cancer and Research Institute (GCRI), B.J. Medical College (BJMC), Ahmedabad, IND

**Keywords:** chemotherapy for pdac and biliary tracts carcinoma, ductal pancreatic carcinoma, folfirinox chemotherapy, gastrointestinal oncology, gemcitabine plus nab-paclitaxel therapy, neo-adjuvant chemotherapy

## Abstract

Background

Pancreatic ductal adenocarcinoma (PDAC) is a highly aggressive and difficult-to-treat cancer, with five-year survival rates still low despite advances in treatment. Borderline resectable pancreatic cancer (BRPC) is associated with a high risk of margin-positive resection and early systemic relapse when treated with upfront surgery alone. Neoadjuvant systemic therapy is therefore recommended to improve resectability and oncologic outcomes. Although combination chemotherapy with 5-fluorouracil (5-FU), leucovorin, irinotecan, and oxaliplatin (FOLFIRINOX) is effective, its toxicity limits use in routine clinical practice. Gemcitabine plus nab-paclitaxel (GnP) represents a commonly used alternative with a more favourable tolerability profile.

Methods

This retrospective audit included consecutive adult patients with histologically confirmed BRPC treated with neoadjuvant GnP at a single tertiary care center between January 2022 and December 2024. Borderline resectability was defined using National Comprehensive Cancer Network criteria, with institutional interpretation informed by international consensus recommendations. Patients received three to six cycles of gemcitabine and nab-paclitaxel according to institutional standards. Radiologic response was assessed using the standard guideline Response Evaluation Criteria in Solid Tumors (RECIST) version 1.1, with qualitative evaluation of the tumor-vessel interface guiding surgical decision-making. Surgical, pathological, toxicity, progression-free survival (PFS), and overall survival (OS) outcomes were analyzed.

Results

Thirty-four patients were included, with a median age of 61 years; 27 patients (79%) had Eastern Cooperative Oncology Group (ECOG) performance status (PS) 1, and seven patients (21%) had ECOG performance status 2. Partial response (PR) was observed in 14 patients (41%), stable disease (SD) in 13 patients (38%), and progressive disease (PD) in 7 patients (21%). Improvement in the tumor-vessel interface was documented in 16 patients (47%). Surgical exploration was undertaken in 22 patients (65%), and curative-intent resection was achieved in 19 patients (56%) of the overall cohort. R0 resection was achieved in 13 (68%) of resected patients. Median PFS was 14.2 months, and median OS was 22.8 months, with a three-year OS of 28%. These outcomes were consistent with previously reported neoadjuvant GnP studies and the Locally Advanced Pancreatic Cancer Cetuximab and nab-Paclitaxel Trial (LAPACT) trial experience.

Conclusions

Neoadjuvant gemcitabine and nab-paclitaxel combination is a feasible and effective treatment option for borderline resectable pancreatic adenocarcinoma in real-world practice. While outcomes may be inferior to those reported with FOLFIRINOX in highly selected patients, GnP provides meaningful disease control with acceptable toxicity and remains a pragmatic option when intensive regimens are unsuitable.

## Introduction

Pancreatic ductal adenocarcinoma (PDAC) is one of the most dangerous and tough to treat solid cancers, with five-year survival rates remaining below 15% despite some of the major advances in surgery, systemic therapy, and perioperative care [1]. A clinically important subset of patients presents with borderline resectable pancreatic cancer (BRPC), defined by limited arterial or venous involvement and associated with a high risk of margin-positive resection and early systemic relapse when treated with upfront surgery alone [1].

Neoadjuvant systemic therapy has therefore emerged as the preferred initial approach for BRPC. This strategy aims to improve resectability, increase the likelihood of achieving an R0 resection, treat occult micro-metastatic disease at an early stage, and allow biological selection by identifying aggressive disease during treatment [2]. Combination chemotherapy with 5-fluorouracil with leucovorin, irinotecan, and oxaliplatin (FOLFIRINOX) has demonstrated substantial activity in pancreatic cancer but is frequently limited in routine practice by toxicity, older age, comorbidities, biliary sepsis, and borderline performance status [3].

Gemcitabine combined with nab-paclitaxel (GnP) represents a widely used alternative regimen, offering clinically meaningful efficacy with a more favorable tolerability profile [4]. Several prospective and retrospective studies have evaluated GnP in neoadjuvant and conversion settings for resectable, borderline resectable, and locally advanced pancreatic cancer, reporting encouraging resection and margin-negative rates [5,6]. However, real-world data from single-institution cohorts remain limited. This retrospective audit evaluates three-year outcomes of neoadjuvant GnP in patients with BRPC treated at a single tertiary care center. The primary objectives were to assess radiologic response, surgical feasibility, pathological outcomes, and survival. Secondary objectives included comparison with published neoadjuvant GnP series and contemporary treatment strategies.

## Materials and methods

Study design and patient selection

This was a retrospective audit of consecutive adult patients with histologically confirmed PDAC classified as BRPC at diagnosis and treated between January 2022 and December 2024. All cases were reviewed by a multidisciplinary tumor board.

Inclusion and exclusion criteria

Patients were eligible for inclusion if they were aged 18 years or older and had histologically confirmed pancreatic ductal adenocarcinoma. All patients were diagnosed with borderline resectable pancreatic cancer at presentation based on National Comprehensive Cancer Network (NCCN) criteria using contrast-enhanced imaging. In addition, patients must have received neoadjuvant chemotherapy with gemcitabine plus nab-paclitaxel at the study center as part of their initial treatment plan. Only patients managed and followed at the same institution during the study period were included in the analysis. Patients were excluded if they had metastatic or clearly unresectable pancreatic cancer at the time of diagnosis. Patients who had received prior chemotherapy or radiotherapy for pancreatic cancer were also excluded. In addition, those with poor performance status who were not suitable for systemic chemotherapy were not included. Cases with incomplete medical records or insufficient follow-up data were also excluded from the analysis.

Definition of borderline resectability

Borderline resectable disease was defined according to National Comprehensive Cancer Network criteria based on contrast-enhanced computed tomography, including limited venous involvement with re-constructable anatomy and/or short-segment arterial abutment without encasement [1]. Baseline staging included cross-sectional imaging and measurement of carbohydrate antigen 19-9, when feasible. International consensus guidelines further informed institutional interpretation of vascular involvement and surgical decision-making [7].

Neoadjuvant treatment protocol

Neoadjuvant chemotherapy consisted of gemcitabine 1000 mg/m² and nab-paclitaxel 125 mg/m² administered intravenously on days 1, 8, and 15 of a 28-day cycle. Three to six cycles were planned based on radiologic response and tolerability. Dose modifications and treatment delays were permitted according to institutional practice [8].

Radiologic and surgical assessment

Radiologic assessment was performed after three to four cycles and at the completion of neoadjuvant therapy. Response was evaluated using RECIST version 1.1. In addition to size-based criteria, qualitative assessment of the tumor-vessel interface was used to guide surgical exploration decisions [9].

Pathological evaluation

Resected specimens were evaluated using the College of American Pathologists tumor regression grading system. Resection margins were classified as R0 or R1, and lymph node status was recorded.

Toxicity and statistical analysis

Treatment-related toxicities were graded using the Common Terminology Criteria for Adverse Events [8]. Progression-free survival (PFS) and overall survival (OS) were estimated using the Kaplan-Meier method.

## Results

Patient characteristics

Thirty-four patients met the eligibility criteria, whose baseline characteristics are given in Table 1. The median age was 61 years, and 20 patients (59%) were male. Tumors were located in the pancreatic head or uncinate process in 26 patients (76%). Venous involvement was present in 29 patients (85%), and limited arterial involvement in 11 patients (32%). An ECOG performance status of 0-1 was observed in 27 patients (79%), and an ECOG performance status of 2 was observed in 7 patients (21%).

**Table 1:** Baseline patient characteristics ECOG: Eastern Cooperative Oncology Group, PS: Performance status, CA19.9: Cancer antigen/Carbohydrate antigen 19.9 (tumor marker).

Characteristic	Value
Number of patients	34
Median age (years)	61 (range 44–73)
Male sex	20 (59%)
ECOG PS 0–1	27 (79%)
ECOG PS 2	7 (21%)
Tumor location (head/uncinate)	26 (76%)
Elevated CA19-9	25 (74%)
Venous involvement	29 (85%)
Arterial involvement	11 (32%)

Neoadjuvant treatment delivery

Neoadjuvant treatment delivery details are given in Table 2. The median number of GnP cycles administered was 4 cycles. 21 patients (62%) completed at least 4 cycles. Dose reductions were required in 13 patients (38%), and treatment delays occurred in 10 patients (29%). Early discontinuation occurred in 7 patients (21%), most commonly due to disease progression or declining performance status (Table 2).

**Table 2:** Neoadjuvant treatment delivery

Parameter	Result
Median cycles received	4
≥4 cycles completed	21 (62%)
Dose reductions	13 (38%)
Treatment delays	10 (29%)
Early discontinuation	7 (21%)

Radiologic response

The radiological response after neoadjuvant therapy is given in Table 3. According to Response Evaluation Criteria in Solid Tumors (RECIST) version 1.1, partial response was observed in 14 patients (41%), stable disease in 13 patients (38%), and progressive disease in 7 patients (21%). Improvement at the tumor-vessel interface was documented in 16 patients (47%), enabling surgical consideration despite limited tumor size reduction in some cases (Table 3).

**Table 3:** Radiological response after neoadjuvant therapy

Response	Patients (%)
Partial response	14 (41%)
Stable disease	13 (38%)
Progressive disease	7 (21%)
Improved vascular interface	16 (47%)

Surgical and pathological outcomes

The surgical exploration and resection outcomes of the study are presented in Table 4. Twenty-two patients (65%) underwent surgical exploration. Curative-intent resection was performed in 19 patients (56% of the overall cohort), while exploration was aborted in three patients (9%) due to unresectable disease. Venous resection and reconstruction were required in 11 patients (58%).

**Table 4:** Surgical exploration and resection outcomes

Outcome	Value
Surgically explored	22 (65%)
Resected	19 (56%)
Exploration aborted	3 (9%)
Vascular resection required	11 (58% of resected)

Pathological outcomes in resected patients are given in Table 5. Among resected patients, major pathological response (College of American Pathologists grade 0-1) was observed in four patients (21%). R0 resection was achieved in 13 patients (68%), and node-negative disease in seven patients (37%).

**Table 5:** Pathological outcomes in resected patients CAP: College of American Pathologists.

Parameter	Result
CAP grade 0–1	4 (21%)
CAP grade 2	9 (47%)
CAP grade 3	6 (32%)
R0 resection	13 (68%)
R1 resection	6 (32%)
Node-negative disease	7 (37%)

Toxicity

Grade 3-4 toxicities were predominantly hematologic, given in Table 6. Neutropenia occurred in six patients (18%), thrombocytopenia in four patients (12%), fatigue in five patients(15%), and peripheral neuropathy in three patients (9%). Febrile neutropenia occurred in two patients (6%), and no treatment-related deaths were observed.

**Table 6:** Grade 3–4 treatment-related toxicities

Toxicity	Frequency
Neutropenia	6(18%)
Thrombocytopenia	4(12%)
Fatigue	5(15%)
Peripheral neuropathy	3(9%)
Febrile neutropenia	2(6%)

Survival outcomes

Survival outcomes are given in Table 7. At a median follow-up of 36 months, median progression-free survival (PFS) was 14.2 months and median OS was 22.8 months. Two-year overall survival (OS) was 44%, and three-year OS was 28% (Table 7).

**Table 7:** Survival outcomes

Outcome	Median
Progression-free survival	14.2 months
Overall survival	22.8 months
2-year overall survival	44%
3-year overall survival	28%

## Discussion

This single-institution audit demonstrates that neoadjuvant GnP provides clinically meaningful disease control in patients with BRPC treated in routine practice. GnP has also demonstrated encouraging activity in the neoadjuvant setting. Retrospective series have reported resection rates of 60-65% and R0 resection rates of approximately 70-75%, supporting its role as a feasible alternative regimen for BRPC [5,6]. Similarly, the LAPACT phase II study evaluating GnP in locally advanced pancreatic cancer reported disease control in 78% of patients and conversion to surgery in about 15-20%, indicating meaningful tumor stabilization with this regimen [10]. These findings support the clinical benefit observed in the present cohort, as shown in Table 8. Slightly lower rates in the present cohort likely reflect inclusion of patients with ECOG performance status 2, frequent dose modifications, biliary complications, and incomplete delivery of planned therapy.

**Table 8:** Comparison with selected published neoadjuvant gemcitabine–nab-paclitaxel studies BRPC: Borderline Resectable Pancreatic Cancer, LAPACT: Locally Advanced Pancreatic Cancer Cetuximab and nab-Paclitaxel Trial, LAPC: Locally Advanced Pancreatic Cancer.

Study	Population	Resection rate	R0 rate	Median OS
Present audit	BRPC	56%	68%	22.8 mo
Published BRPC series	BRPC	~60–65%	~70–75%	~24–26 mo
LAPACT trial	LAPC	Conversion subset	NA	~18–19 mo

Radiologic response does not always correlate with surgical outcomes in pancreatic cancer. Several studies have demonstrated that RECIST-based tumor size reduction occurs in fewer than 30% of patients, yet a larger proportion can still undergo successful resection due to improvements in tumor-vessel interface and treatment-induced fibrosis [9,11]. Pathological response after neoadjuvant therapy is increasingly recognized as an important prognostic marker. Reports from neoadjuvant GnP studies have documented major pathological response rates of approximately 15-25%, which are associated with improved postoperative outcomes and survival [5,10]. The rate observed in this study is therefore consistent with previously published data.

Toxicity profiles observed with GnP are also consistent with prior studies. In metastatic pancreatic cancer, the Metastatic Pancreatic Adenocarcinoma Clinical Trial (MPACT) demonstrated improved overall survival with GnP (8.5 months vs. 6.7 months with gemcitabine alone) with manageable toxicity, including grade ≥3 neutropenia in about 38% of patients and febrile neutropenia in <5% of patients [4,8]. Compared with FOLFIRINOX, which improved median survival to 11.1 months versus 6.8 months with gemcitabine but is associated with higher rates of grade 3-4 toxicity, GnP remains a practical option for patients with borderline performance status [3,12].

Limitations

The study has several limitations. It is a retrospective review from a single center with a small number of patients, so the findings may not apply to all settings. There was no comparison group, and treatment choices and imaging assessments varied between patients. Follow-up was limited, so longer-term outcomes could not be fully assessed.

## Conclusions

In this three-year single-institution audit, the neoadjuvant gemcitabine and nab-paclitaxel combination therapy was feasible and effective in patients with borderline resectable pancreatic adenocarcinoma, enabling curative-intent resection in over half of the cohort. Outcomes were consistent with real-world experience and support GnP as a pragmatic neoadjuvant option when FOLFIRINOX is unsuitable. Prospective studies are needed to optimize patient selection and treatment sequencing.
